# Lipid-Based Nanocarriers for Ophthalmic Administration: Towards Experimental Design Implementation

**DOI:** 10.3390/pharmaceutics13040447

**Published:** 2021-03-26

**Authors:** Felipe M. González-Fernández, Annalisa Bianchera, Paolo Gasco, Sara Nicoli, Silvia Pescina

**Affiliations:** 1 Department of Food and Drug, University of Parma, Viale Parco Area delle Scienze, 27/a, 43124 Parma, Italy; annalisa.bianchera@unipr.it (A.B.); sara.nicoli@unipr.it (S.N.); 2 Nanovector S.r.l., Via Livorno, 60, 10144 Torino, Italy; paolo.gasco@nanovector.it

**Keywords:** design of experiments, optimisation, ocular delivery, solid lipid nanoparticles, SLN, nanostructured lipid carriers, NLC, microemulsion, quality by design, factorial design

## Abstract

Nanotherapeutics based on biocompatible lipid matrices allow for enhanced solubility of poorly soluble compounds in the treatment of ophthalmic diseases, overcoming the anatomical and physiological barriers present in the eye, which, despite the ease of access, remains strongly protected. Micro-/nanoemulsions, solid lipid nanoparticles (SLN) or nanostructured lipid carriers (NLC) combine liquid and/or solid lipids with surfactants, improving drug stability and ocular bioavailability. Current research and development approaches based on try-and-error methodologies are unable to easily fine-tune nanoparticle populations in order to overcome the numerous constraints of ocular administration routes, which is believed to hamper easy approval from regulatory agencies for these systems. The predictable quality and specifications of the product can be achieved through quality-by-design (QbD) implementation in both research and industrial environments, in contrast to the current quality-by-testing (QbT) framework. Mathematical modelling of the expected final nanoparticle characteristics by variation of operator-controllable variables of the process can be achieved through adequate statistical design-of-experiments (DoE) application. This multivariate approach allows for optimisation of drug delivery platforms, reducing research costs and time, while maximising the understanding of the production process. This review aims to highlight the latest efforts in implementing the design of experiments to produce optimised lipid-based nanocarriers intended for ophthalmic administration. A useful background and an overview of the different possible approaches are presented, serving as a starting point to introduce the design of experiments in current nanoparticle research.

## 1. Introduction

Recent estimations account for 2.2 billion people being globally affected by vision impairment or blindness. Current lifestyles regarding dietary habits or sedentarism, combined with a constantly ageing population, forecast an increase in chronic ocular diseases such as glaucoma (from 76 to 95.4 million) or age-related macular degeneration (from 195.6 to 243.3 million) for 2030. Furthermore, ocular infections might also lead to visual impairment, especially if left untreated [1].

To date, several drug classes have been investigated to treat these conditions, but their efficacy is influenced by effective ocular delivery, which remains a challenging task [2]. In fact, despite being easily accessible, the eye preserves its integrity by constantly hindering the access of xenobiotics, including drugs, through a series of static, dynamic and metabolic barriers [3].

Over the past decades, major advances in nanotechnology have established nanomedicine as a promising tool for ophthalmic drug delivery improvement [4]. Nanocarriers allow an increase in drug solubility and stability by encapsulation in nanometric structures (20–1000 nm) with a high surface area, which ultimately results in improved bioavailability at different ocular targets [5,6]. Particularly, lipidic nanocarriers such as solid lipid nanoparticles (SLN) or nanostructured lipid carriers (NLC) show increased eye compatibility since they are composed of highly biocompatible and naturally occurring lipids [7,8]. In a similar manner, lipid matrices also allow for better solubilisation and protection of hydrophobic compounds, and in combination with surfactants, they increase the apparent drug solubility in aqueous media, which are preferable vehicles for ocular administration [9].

Effective development of colloidal drug carriers intended for ocular delivery remains a cumbersome task [10,11]. Quality by design (QbD) proposes the implementation of systematic approaches, rather than classical empirical methodologies, for effective patient-oriented product development [12]. Several efforts have been made to formulate nanoparticles following a more structured approach based on a statistical design of experiments (DoE), one of the pillars in QbD [13]. This multivariate approach creates mathematical relationships between operator-controllable inputs of the production process and final qualities of the product. As a result, a better understanding of the process is achieved and optimal products with target characteristics can be obtained [14].

Quality by design and thus design of experiments have already been widely applied in the food or chemistry industries for product optimisation and process improvement [15,16,17]. Applications in the pharmaceutical field have been constantly growing in the past years: from solid dosage forms [18] to nanoparticulate systems such as liposomes, polymeric micelles or drug nanocrystals [19,20,21]. The number of specification requirements in a formulation intended for ocular administration can be demanding [22]. Considering the potential benefits of colloidal lipid-based systems in improving ocular drug bioavailability, some authors have proposed the development and optimisation of these carriers following a statistical design of experiments.

This review aims to present a compilation of the latest efforts toward the implementation of experimental designs in the optimisation of ophthalmic lipid-based nanocarriers. In the first part, a general overview of the available ocular administration routes is dis-cussed, followed by a description of some lipid-based nanocarriers’ characteristics. In the second part, the novel quality-by-design production approach is introduced and an ex-planation of the bases of the design of experiments is provided. This information will help to further analyse the recent publications available in this research area. To the best of our knowledge, no review of this kind has yet been proposed, and the present paper is intend-ed to serve as an initial simplified starting point for approaching the fascinating topic of statistical design of experiments.

## 2. Overcoming Ophthalmic Barriers with Lipid-Based Nanocarriers

### 2.1. Ocular Anatomy and Drug Delivery Approaches

**Figure 1:**
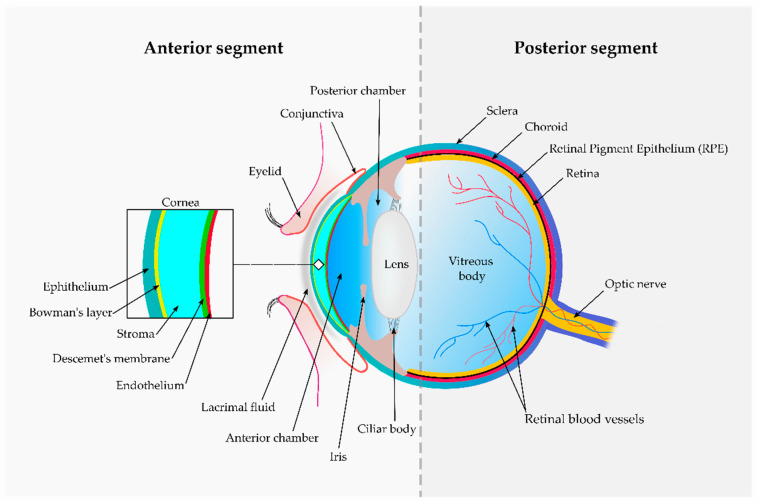
Schematic representation of the ocular anatomy. An imaginary line (dotted grey vertical line) divides the ocular structures into two segments. The anterior segment contains the cornea (detailed within the insert), iris, ciliary body and both anterior and posterior chambers. The aqueous humour fills the anterior and posterior chambers, while most of the posterior segment is filled by a hyaluronan-rich gel known as vitreous humour. The posterior segment also contains the sclera, similar in composition to the corneal stroma. The choroid is a highly vascularised tissue separated from the retina (neural tissue) by a monolayer of hexagonal pigment-containing cells: the retinal pigment epithelium.

The eye is one of the most sophisticated human organs. It has become commonplace to distinguish two ocular segments: the anterior segment, which is mostly exposed and visible, and the posterior segment, inside the ocular orbit, which includes important structures such as the retina, a neural tissue (Figure 1). Based on the target segment, several administration routes are available [3].

Topical administration commonly addresses diseases affecting the anterior segment of the eye, such as keratitis, conjunctivitis or glaucoma. Eye drops account for 70% of prescriptions due to their ease of use, high patient acceptability and cost-effectiveness [23]. The ocular surface is covered by 7–9 µL of lacrimal fluid, which presents an external lipidic layer and two consecutive hydrophilic layers with a predominantly aqueous and mucous nature, respectively. Administration of a single formulation drop activates reflex blinking and is quickly washed away through nasolacrimal drainage and tear turnover. Nonspecific absorption trough the conjunctiva, which covers the eye surface and the interior of the eyelids except the cornea (Figure 1), further reduces the remaining formulation available for transcorneal absorption [24].

The cornea, as detailed in Figure 1, is a highly differentiated tissue containing a lipophilic external epithelium and an internal endothelium separated by a thicker hydrophilic stroma [25,26]. This hydrophobicity gradient and an elevated presence of inter-cellular tight junctions within the epithelium hinder simple drug diffusion, which consequently relies on substance molecular weight and the hydrodynamic radius [2]. Less than 5% of the initially applied dose reaches the aqueous humour in the anterior chamber (Figure 1). In this region, several convective flows such as the aqueous humour circulation or the uveal turnover further reduce drug availability [27]. Unfortunately, the anterior segment does not generally benefit from systemic administration that, on the contrary, could eventually reach the posterior segment. However, the observed drug levels after oral or intravenous administration are normally non-productive due to the presence of two blood–retinal barriers. The first, known as the outer blood–retinal barrier, is mainly composed of the retinal pigmented epithelium: a melanin-rich monolayer, intimately adhered to the fenestrated choriocapillaris and able to restrict drug distribution into the retina (Figure 1). The second, known as the inner blood–retinal barrier, is composed of fine capillaries embedded in the retina, presenting a tightly closed endothelium that impedes drug access through the paracellular route [28]. Despite these barriers, some treatment options relying on intravenous administered drugs, such as photodynamic therapy, are used in current clinical practice [29].

Intravitreal injections directly address the posterior segment of the eye, targeting the vitreous body and, ultimately, the retinal region, where degenerative diseases such as diabetic retinopathy and age-related macular degeneration exert their damage [30]. Both low-molecular-weight compounds (i.e., corticosteroids) and high-molecular-weight compounds such as biologicals (i.e., bevacizumab, ranibizumab, pegaptanib) are currently administered by intravitreal injection. Multiple side effects due to the invasiveness of the procedure have been reported, such as endophthalmitis, cataract or retinal detachment [31,32,33]. Diffusion in the vitreous body is strongly dependent on the molecule hydrodynamic radius and the presence of a surface charge, which also determines the residence time. Large hydrodynamic radii hamper drug mobility, while the presence of cationic surface charges allows for interaction with the negatively charged collagen and hyaluronan present in the vitreous humour, increasing in both cases drug permanence and reducing dosing frequency [34]. Elimination occurs towards the anterior segment (aqueous humour) through the blood–retinal barriers and, to a lesser extent, by biotransformation. As a result, small lipophilic molecules show half-life values of several minutes, while larger molecules (i.e., proteins) remain up to several days [28,35].

Recent approaches based on periocular administration routes (subconjunctival, sub-Tenon’s, retrobulbar, peribulbar and intrascleral) are less invasive than intravitreal injections, respecting the immunological privilege of the inner eye and allowing for formulation deposition at different locations of the posterior segment [36]. Drug absorption occurs via the trans-scleral pathway and faces three static barriers, namely sclera, choroid and retinal pigmented epithelium (RPE) [37,38]. The sclera, similar in composition to the corneal stroma, with a high water and collagen content, shows better permeation to hydrophilic, neutral or negatively charged molecules with low molecular radii. Similar molecular properties have demonstrated to offer better permeation across Bruch’s membrane (the innermost layer of the choroid) and the choroid itself. On the other hand, the RPE favours the passage of more lipophilic molecules [39,40]. In addition, several dynamic barriers (i.e., conjunctival, episcleral or choroidal clearance and uveoscleral outflow) and metabolic barriers (mainly cytochrome P-450 and lysosomal enzymes in the RPE) further impede drug access to the neuroretina [41].

Thus far, although extensive research has been carried out, good ocular bioavailability remains a challenge, especially in the most patient-friendly topical administration route [42].

### 2.2. Improving Drug Access to Ocular Structures with Lipid-Based Nanocarriers

Nanotechnology has seen an exponential growth in the past decades, leading to the development of multiple versatile drug carriers in the range of 10–1000 nm, such as liposomes, polymeric micelles or solid lipid nanoparticles [43]. Encapsulation into nanoparticles with a high specific surface area allows for increased drug stability and solubility, while maintaining a low side-effect profile [44].

Poorly water-soluble drugs with a strong therapeutic potential have especially benefited from nanoencapsulation, being postulated as a promising tool for circumventing the various ocular barriers. In fact, extensive research in the field has demonstrated an in-crease in drug bioavailability and residence time at ocular target tissues [45]. As a result, some products are already present in the market, such as Restasis^®^ [46], Ikervis^®^ [47] or Cequa^®^ [48].

**Figure 2:**
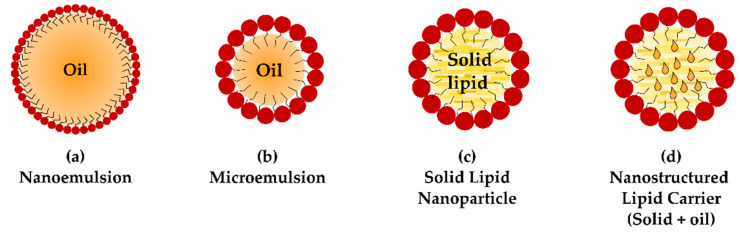
Schematic representation of different lipid-based nanocarriers. Nanoemulsions (**a**) and microemulsions (**b**) both present a liquid oily core but differ in their thermodynamic stability, the former being subject to typical emulsion instabilities, while the latter are thermodynamically stable. Solid lipid nanoparticles (SLN) (**c**) present a rigid lipid matrix with poor drug-loading capability, which is improved by partial substitution with an oil, leading to the so-called nanostructured lipid carriers (NLC) (**d**).

During the end of the last century, several nanoparticulate systems based on lipid matrices (also termed lipid-based nanocarriers) have emerged, such as micro- or nanoemulsions, solid lipid nanoparticles (SLN) and nanostructured lipid carriers (NLC) (Figure 2).

Compared to other extensively studied nanocarriers based on polymers, their production methods usually avoid the presence of residues from organic solvents and allow for easy scale-up manufacturing. Moreover, the physiological lipids that form the matrix commonly benefit from the denomination of Generally Recognized as Safe (GRAS) from the regulatory agencies [49,50]. When applied topically, for example, the lipid core is believed to act as a drug depot in the lipid layer of the tear film, while the presence of a non-ionic surfactant is demonstrated to enhance corneal penetration [7]. The low nanoparticle size also accounts for the translucent/transparent appearance to the naked eye, increasing patient acceptance. Regarding the formulation, it has been reported that a size reduction under 90 nm allows for better stability of the colloidal dispersion since gravitational phase-separation is prevented by Brownian motion [51,52].

Pseudo-ternary mixtures of a lipid, surfactant/cosurfactant and water can lead to multiple systems, depending on the relative proportions of each component, their chemical nature and/or the processing parameters. Well-known systems such as oil-in-water (o/w) or water-in-oil (w/o) emulsions or micro-/nanoemulsions can be obtained. In a similar manner, normal or reverse micelles and more complex systems such as bicontinuous or mesomorphous phases have also been reported [53,54].

Oil-in-water nanoemulsions are colloidal dispersions of an oil, stabilised with a surfactant, that appear as nanometric spheres with a typical nanoparticle size under 100 nm [52]. They are obtained by means of high mechanical shear, remaining as a thermodynamically unstable colloid. The colloidal state shows a higher free-energy value than the separated-phase state, and only a small activation energy barrier (ΔG*) avoids formulation breakdown by diverse phenomena: flocculation, coalescence, gravitational separation and/or Ostwald ripening [55]. Their kinetic stability strongly depends on the balance between forces that promote particle interaction (i.e., Brownian or gravitational forces) and repulsive steric and/or electrostatic forces. Nonetheless, it has been reported that a metastable phase of weeks or even months can be achieved by several approaches. Proper selection of a particle size distribution under 90 nm has been demonstrated to avoid creaming phenomena. Similarly, a lipidic phase with low water solubility prevents Ostwald ripening, and the addition of stabilisers (texture modifiers, ripening retarders) prevents flocculation and coalescence phenomena [56]. Poorly water-soluble drugs such as dorzolamide, terbinafine or acetazolamide have already been formulated as ophthalmic nanoemulsions with positive results [57,58,59]. A clobetasol propionate nanoemulsion for inflammation after cataract surgery is already in a phase III trial [60], while, as mentioned above, cyclosporine [46,47] and difluprednate [61] nanoemulsions are already on the market.

Different from nanoemulsions, microemulsions exhibit thermodynamic stability and reduced nanoparticle average sizes, appearing optically transparent. In fact, oil-in-water microemulsions are defined as lyotropic phases obtained by spontaneous self-assembly and not by mechanical shear, resulting therefore in a thermodynamically stable system [52]. In this case, the free energy of the colloid is lower than the separated-phase state due to an increased surfactant-to-oil ratio. An ultra-low interfacial tension is achieved, and multiple spheroid-like shapes can be observed since colloid nanoparticles do not require to be spherical in order to further reduce interfacial tension [55,62,63]. Though the formation will theoretically occur spontaneously, a small energy input helps overcome kinetic energy barriers and mass transport limitations. The system remains unaltered if no chemical and/or microbiological degradation occurs, and is reversible after agitation or heating/cooling processes [64]. Multiple ocular therapeutics have been loaded into microemulsion systems, such as cyclosporine, dexamethasone or pilocarpine [65].

Lipids that appear solid at room temperature can also emulsify if heated over their melting point, while further solidification of these globules can lead to the formation of the so-called solid lipid nanoparticles (SLN). Several production methods have been proposed, including high-pressure homogenisation followed by sonication or melt emulsification [50]. SLN present a solid lipid core that protects chemically labile molecules and allows for the possibility of controlled release of both lipophilic and hydrophilic drugs, maintaining a good cost-effectiveness profile [66,67]. Nonetheless, some drawbacks such as high water content (77–99%), poor drug loading and early drug expulsion have been reported [50].

Solid lipid matrices typically show different polymorphic transitions over time [68]. As a result, densely packed crystalline lattices are progressively formed, and drug interpenetration between fatty acid chains is hindered, leading to premature expulsion [69]. Non-homogeneous matrices that combine long- and medium-chain acylglycerols have been demonstrated, for instance, to increase drug-loading capacity by creating spaces between the different-length fatty acid chains [70,71].

Modifying the lipid polymorphism, and thus improving drug stability, can also be achieved by addition of a liquid lipid to the solid matrix, leading to the so-called nanostructured lipid carriers (NLC) [72,73]. It has been postulated that the added oil can remain as small droplets that solubilise most of the drug and are protected by the surrounding solid matrix (known as multiple-type NLC). In other cases, depending on oil and solid lipid miscibility, a new amorphous matrix with improved polymorphic behaviour is believed to be formed (amorphous-type NLC) [74]. The number of therapeutics that have been successfully incorporated into SLN and NLC has grown in the past decades and cover several ocular disorders such as inflammation (indomethacin, sodium diclofenac) or infections (ofloxacin, azithromycin, acyclovir or ketoconazole). Comprehensive compilations can be found elsewhere [8,75].

In summary, drug incorporation into nanoparticulate systems based on lipid matrices has demonstrated to increase formulation mucoadhesivity, thus improving transcorneal diffusion and cellular uptake if applied topically [9,76]. Nonetheless, some limitations have been identified regarding the ability to obtain consistent nanoparticle populations. The relatively simple manufacturing processes and the low number of excipients required contrast with the high sensitivity of the production process to small variations. In fact, Bastogne recently proposed simple calculations that demonstrate the elevated number of possible different nanoparticles that can be obtained from a single set of components and a single production process [13]. In addition, when the ocular administration route is considered, new challenges arise in the development of efficient nanoformulations. Precise control of key parameters such as average size, polydispersity or surface charge becomes fundamental in order to effectively overcome physical and mechanical barriers. At the same time, a compromise between colloidal system stability, ocular compatibility (pH, osmolarity) and excipient toxicity must be achieved [77].

Many scientists hold the view that identification of critical operator-controllable in-dependent variables could help overcome this variability issue [78]. Multivariate analysis tools, based on statistics, allow to understand the simultaneous contribution of various factors in altering final formulation characteristics [79]. The development of mathematical models that predict nanoformulation specifications has been proposed as a valuable tool for nanoformulation optimisation [80,81,82]. Recent research suggests that ophthalmic drug delivery could especially benefit from this ability to fine-tune nanocarrier characteristics, not only from a therapeutic point of view, but also considering a regulatory framework.

## 3. Optimising the Outcomes: Statistical Design of Experiments (DoE)

### 3.1. Quality by Design (QbD)

The relative low availability of marketed products containing nanoparticulate systems, despite the strong research efforts of the last decades, is mainly considered to rely on the inability to control their quality and safety [83]. During nanotherapeutics production, small variations in the manufacturing process can result in significant deviation from the quality attributes required for the nanoparticle population. In addition, a lack of adequate regulatory and safety guidelines has restrained manufacturers from exploring this field, and thus, every market regulation attempt requires a case-by-case approach [84].

The classical quality-by-testing (QbT) regulatory framework ensures final product adequacy through inflexible manufacturing steps and extensive testing on bulk materials and both intermediate and final products. Batch quality failure is usually not clearly understood, nor investigated for its cause. After market approval, any further modification requires extensive regulatory supplements, which hampers easy process evolution and adaptability. With the expected growth in the prevalence of ocular chronical diseases, new approaches are required to facilitate market access of valuable candidates based on nanotechnology [85].

In the last years, regulatory agencies such as the European Medicines Agency (EMA) and the U.S. Food and Drug Administration (FDA) have started to recommend a change in classical quality assurance methods, moving forward to the so-called quality by design (QbD). This term has been defined by the International Conference on Harmonization in its guidelines as ‘a systematic approach to development that begins with predefined objectives and emphasizes product and process understanding and process control, based on sound science and quality risk management’ [86].

In opposition to traditional systems, QbD allows to unveil the relationships between manufacturing variables and critical, patient-oriented quality product attributes. The production remains consistent and robust but is also flexible to changes. In fact, end-product testing becomes almost secondary since process control and exhaustive identification of possible variability sources are the actual quality assurance. As a result, batch release may be quicker since constant testing might not be required [85].

Product research and development following QbD starts with a clear definition of the target product (also defined as the quality target product profile (QTPP)) and its critical quality attributes (CQAs). It is of utmost importance, therefore, to identify characteristics that are relevant for the patient and translate them into formulation attributes. Early adoption of a QbD perspective, also in initial research stages, already sets the first steps to a successful patient-focused product. The identification of critical material attributes (CMAs) of bulk materials and critical process parameters (CPPs) allow to control key parameters of the production process that, through proper adjustment, can lead to the de-sired product. All possible sources of variability should be under control, and the risk assessment (RA) activity at the beginning and end of the process allows for systematic identification of possible hazards and the risks associated with them [14].

Quality-by-design approaches increase research efficacy, benefiting both manufacturers and regulatory agencies. The former reduces the time and cost of research, and the latter benefits from a wider and more robust understanding of the production process that could ease market approval of complex nanoparticulate systems [87]. Patients also benefit from QbD since a new generation of therapeutics, based on nanocarriers, could easier access commercialisation, improving actual therapeutic options. As a result, early adaptation of QbD approaches might lead to effective translation of years of research into innovative marketed nanotherapeutics.

### 3.2. Design of Experiments (DoE)

Assessing the influence of CMAs and CPPs affecting the final product can be per-formed using univariate or multivariate analysis techniques.

In univariate analysis, all factors are set at a baseline level, and each time, a single factor is changed to different levels, analysing the result of this change on the final product. This classical one-factor-at-a-time plan eventually identifies the most important factors (or *main effects*) but fails to account for possible synergisms/antagonisms between the factors (*factor interactions*). In addition, the best factor setting that leads to an optimal product is not guaranteed to be properly elucidated. A more efficient approach to unveil these critical interactions, of utmost interest within the QbD framework, is the so-called statistical design of experiments (DoE) [88].

Design of experiments is a multivariate statistical tool that allows one to identify relationships between factors influencing a process and the observed outputs. In addition, DoE helps to identify optimal process conditions and the design space. The International Council for Harmonisation of Technical Requirements for Pharmaceuticals for Human Use (ICH) guidelines define the design space as ‘the multidimensional combination and interaction of input variables (e.g., material attributes) and process parameters that have been demonstrated to provide assurance of quality’. Therefore, changes within this design space would not require any regulatory post-approval evaluation, which could potentially benefit nanoparticulate systems once on the market [85]. This is accomplished through a series of predefined and structured experiments (or *runs*) by which different combinations of the input variables of interest are explored, recording the observed changes on measurable characteristics of the final product (*responses*). A statistical analysis of the obtained results allows one to develop mathematical equations that describe the contribution of each variable to the changes observed in the response under study [89].

In the context of a production process, CMAs and CPPs are independent variables denoted as *factors* and can have a quantitative or qualitative nature. Quantitative factors are continuous and thus are set within a numerical range (i.e., concentration of an excipient or homogenisation temperature). Discrete variables, such as lipid nature or type of supplier, can also be considered, accounting for categorical factors. The response outputs are selected among critical quality attributes (CQAs) of the final product [90].

Process variability due to uncontrollable nuisance factors that still affect the response should also be considered. Three fundamental tools bring about a bias reduction due to experimental noise: randomisation, blocking and replication. Randomisation of experimental runs contributes to an even distribution of possible experimental errors amongst the measured responses and neutralises time-related effects. Then, identified sources of variation that can be controlled (i.e., operator, ambient temperature or raw material batches) can be minimised by adequate blocking of the experimental runs. Finally, replication of experimental runs gives an estimation of experimental errors and increases the degrees of freedom of the design. The number of statistically fair comparisons that can be established in a set of data increases with increasing degrees of freedom [90,91].

The measured response (y) is, consequently, the overall sum of the effects due to single factors (main effects), interactions between the factors and the experimental noise. The resultant regression model equation considering, for example, two quantitative factors (i.e., A and B) can be expressed according to the general formula

(1) $${y\;=\;\beta }_{0}{\;+\;\beta }_{1}x_{1}{\;+\;\beta }_{2}x_{2}{\;+\;\beta }_{12}x_{1}x_{2}\;+\;\epsilon$$

where x_1_ represents factor A, x_2_ represents factor B and x_1_x_2_ represents the two-factor interaction (AB). The ϵ term represents the experimental error, and the beta parameters (β_1_ and β_2_) are the coefficients to be determined through the experimental design. The existence of the β_12_x_1_x_2_ term indicates that factors A and B contribute in a synergistic (if the sign is positive) or antagonistic (if the sign is negative) manner to modify the response [92].

**Figure 3:**
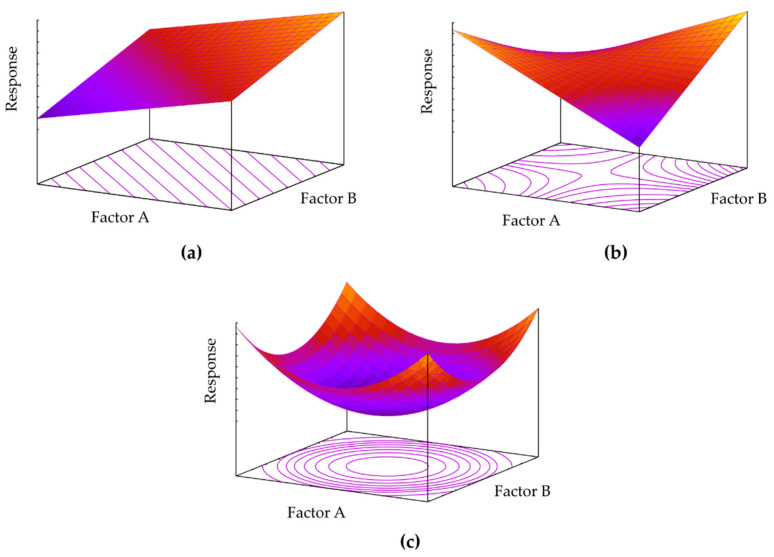
Three-dimensional response surfaces and their respective bidimensional contour plots following (**a**) a linear or first-order function and (**b**) a second-order equation. Inclusion of quadratic terms in the function allows to detect maximums or minimums of the response (**c**). Models calculated with Gnuplot software.

Response equations can be graphically represented creating 3D plots and their respective 2D contour plots (Figure 3). The simplest linear, first-degree polynomial is depicted in Figure 3a. In this way, it is easy to determine how a change in either factor A or factor B produces a change on the response surface. Progressive inclusion of higher-order terms into the polynomial leads to model curvature (Figure 3b,c). For optimisation purposes, usually quadratic (Figure 3c) or rarely cubic terms might need to be included to find a proper fit of the model to the observed response. If more than two factors are studied, graphical representation can only be achieved by fixing a constant value for the other factors studied [92,93].

Getting more into the topic of the review, multiple factors can be considered to affect final lipid-based nanoparticle characteristics. Typical factors regarding CMAs are, for instance, lipid and/or surfactant nature and/or their concentration, while CPPs usually under study are working temperature and/or parameters regarding the homogenisation process [87].

During the initial planning stages, a wide number of factors should be suspected of contributing to response modification. It is important to underline that, nonetheless, following the Pareto rule, only 20% of the factors are considered to contribute to 80% of the observed output. Different approaches allow to select these “vital few” factors (usually 2–4) from the initially considered “trivial many” factors [94]. Factor screening for detection of these vital few factors is sometimes performed following a classical try-and-error methodology, but a special group of experimental designs, known as screening designs, offers a quicker and more efficient approach with low experimental work. Their reduced experimental workload comes with the drawback of limited statistical power: while screening designs can estimate active main effects, they fail to correctly assess factor interactions and model curvature [90,95].

Estimating non-linear effects requires studying at least three levels for each factor, augmenting the number of experiments required. Optimisation experimental designs are also known as response surface methodologies (RSM) and create higher-order model equations that maximise product similarity to the desired CQAs. Therefore, it is important to select a low number of factors under study, so the optimisation step maintains an affordable experimental workload [96,97]. However, recent research in the field has resulted in the appearance of the so-called *definitive* screening designs, which are able, under specific circumstances, to identify active quadratic terms with minimal experimental workload [98].

Considering ocular drug delivery, the mean nanoparticle size (Z-average value) and polydispersity index (PDI) are typical CQAs found to be optimised in experimental designs [99]. Generally, particles of ≤200 nm are considered to offer adequate permeation and mobility through ocular barriers, while small particles, around 20 nm, are quickly cleared out, as demonstrated in periocular administration [36,100]. Therefore, populations with a narrow particle size distribution (PDI < 0.2) and a Z-average value under 200 nm are also desired. The zeta potential accounts for the degree and sign of electrostatic forces between nanoparticles, which affect their stability and aggregation behaviour. Regarding colloidal stability, high absolute values (approx. ±20 mV) are of interest, since lower values might be overcome by attractive forces between particles, leading to formulation instability [101,102]. In addition, following a topical administration route, the presence of a cationic surface charge is believed to improve residence time by interaction with the negatively charged corneal epithelium and the mucins from the tear fluid and the conjunctiva [103,104,105]. High drug loading and entrapment efficiency are also desirable variables to be optimised through DoE. The former accounts for the maximal percentage of a drug that can be encapsulated into the nanoparticles, while the latter quantifies the efficiency of this loading process during formulation production [87].

CPPs can also be considered in the experimental design. In this sense, some authors have included both the number of high-pressure homogenisation cycles and the pressure at which they are performed as variables under study [106]; other authors have included the storing temperature since it might modify long-term stability [107].

### 3.3. Two-Level Factorial Designs

**Figure 4:**
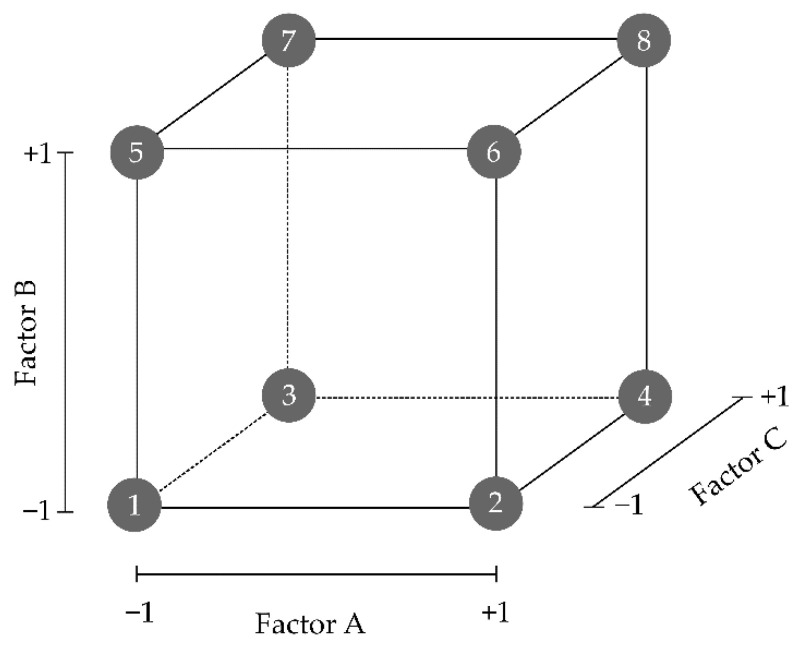
A geometric representation of the eight runs (dark spheres at vertices 1 to 8) of a three-factor, two-level (2^3^) full factorial design. Each factor (A, B and C) is established at two levels, coded as −1 (low level) and +1 (high level). Note that each experimental run (vertices of the cube, numbered 1 to 8) is a unique combination of the different levels for the factors.

Two-level factorial designs are of use when only two values for each factor are studied. Considering *k* = the number of factors studied, 2^k^ possible combinations between factors and levels are explored. As an example, if three factors are taken into consideration (*k* = 3), the number of possible combinations is eight and so are the number of experiments (or runs) that can be performed (Figure 4) [90].

Full factorial experimental designs are a very cost-effective solution when a low number of factors is considered. All possible combinations between the considered factors and levels are studied, maximising the amount of information that can be obtained from an experimental effort. Augmenting the number of factors under study will lead to an exponential increase in the required experimental runs. In several cases, due to time or economic limitations, performing all the experimental runs can become impractical [108]. In this case, a fractional factorial design can be postulated, where only a subset of the experimental runs is executed, while maintaining good predictability. Since only a subgroup of the runs is executed, only a subset of the equation terms can be properly estimated. The remaining unstudied terms are said to be *aliased* or *confounded* amongst themselves. The simplified regression model is still acceptable, considering several statistical principles. As an example, the “hierarchy principle” hypothesises that high-order terms of the equation (three-factor interactions and higher) are supposed to contribute in less magnitude to the observed response than the main effects (i.e., βx₁ and βx₂ terms in Equation (1)) and two-factor interactions (i.e., β₁₂x₁x₂ term in Equation (1)) [109,110,111].

The term *resolution* of fractional designs quantifies the level of aliasing present in a model. Screening designs are not intended to give an exact description of the response but only to help identify the active main effects and two-factor interactions that will be further studied in a consecutive optimisation design, where other high-order interactions can also be properly estimated. Therefore, aiming to reduce the experimental workload, a higher level of aliasing is usually accepted in screening designs. In this sense, Resolution IV (Res IV) fractional factorial designs require a minimum of runs, while still being able to properly identify the active main effects, but two-factor interactions appear confounded between themselves and thus cannot be elucidated. If enough resources and time are available, more experimental runs can be considered in order to create a Resolution V fractional factorial design. This design will also identify active two-factor interactions since they are confounded with higher-order terms but not amongst themselves. Resolution does not apply to full factorial designs, since all the possible factor combinations are under study and no aliasing or confounding is present [89,112].

In addition to the experimental runs situated on the vertexes of the design space (Figure 4), some centre-point experimental runs, with intermediate values between the levels chosen for each quantitative factor, should also be included in the study. These checkpoints provide an estimation of process stability and variability and, thereupon, should not be randomised but evenly distributed at the beginning, intermediate and end of the experimental process. When only two levels of each factor are studied, centre points also help detect the possible curvature of the model, although they fail to properly quantify it [89].

Full factorial designs do not require intensive statistical analysis, and thus, they are an accessible first approach to design of experiments, and some examples have been recently reported to prepare ophthalmic lipid-based nanocarriers (Table 1).

Particularly, Kiss and colleagues [113] obtained dexamethasone-loaded nanostructured carriers intended for topical administration. A high drug entrapment efficiency was achieved (approx. 88%) through drug solubility in lipid-screening tests, followed by crystallinity studies (differential scanning calorimetry (DSC)) of the matrices, which allowed authors to select optimal components for NLC formulation. In a second step, the concentration of the drug, the surfactant and the total lipid amount were selected as independent variables to be studied at two levels in a 2^3^ full factorial experimental design with four responses to be optimised. All the selected variables, especially surfactant concentration, showed a contribution to particle size, while lipid concentration did not affect the entrapment efficiency. The best formulation significantly improved dexamethasone solubility and ensured a high corneal retention of the drug when tested ex vivo. Based on this promising data, the same research group proposed, in a successive publication [114], similar NLC functionalised with a mucoadhesive polymer (hydroxypropyl methylcellulose) that could improve nanoparticle adhesion to the mucosal surface. Two further responses related to nanoparticle population distribution by volume were included in a three-factor, two-level full factorial design that led to eight unique formulations (Table 1). Further studies on human corneal epithelial cell layers demonstrated good biocompatibility, while a depot-like effect on the corneal surface was observed in an ex vivo corneal porcine model, mainly explained by improved mucoadhesivity.

González-Mira and colleagues [107] developed flurbiprofen-loaded NLC through a 2^4^ full factorial design. In addition to the three CMAs included as factors under study (Table 1), a fourth factor, namely storage temperature (either at room temperature or at 4 °C), was included as a CPP, and their influence on the particle mean size, PDI and zeta potential was assessed. The optimised formulation demonstrated good physicochemical stability and high tolerance for eye instillation on both in vitro and in vivo assays.

These examples should be addressed with caution since the authors have studied the quantitative factors at only two levels, inevitably forcing the response to follow a linear model (Figure 3a,b). Inclusion of a few checkpoint experimental runs at intermediate values would have identified a possible curvature of the response, indicating whether further investigation is required. In fact, should the intermediate point response values coincide with the mean value of the two responses at the vertexes, the lowest-order model has been found and no further experimental designs are required. If the experimental response values at these intermediate points (usually denoted as ‘0′ points) differ significantly, a further experimental design with at least three levels for each factor is required in order to adequately model the response behaviour.

### 3.4. Plackett–Burman Designs

Plackett–Burman experimental designs, first introduced in 1946, represent a very economical option for screening a large number of factors [115]. The number of experiments to perform corresponds to the first multiple-of-four higher than the number of factors to be screened. As an example, for eleven factors set at two levels each, a full factorial design would require 2^11^ = 2048 experimental runs, while Plackett–Burman designs with only twelve experimental runs (plus centre points and replicates) are able to adequately estimate main effects. Consequently, this design presents a high level of confounding since it considers negligible all two-factor interactions, and therefore, they are strongly aliased with the main effects, resulting in a Resolution III design [91].

Plackett–Burman experimental designs are only of use in screening stages, usually followed by a second experimental design with a higher resolution (V or more) that permits a more precise main effect estimation and factor interaction inclusion into the mod-el. An example of this application was provided by Rathod and co-authors, who recently reported the development of ibuprofen-loaded NLC intended for topical administration. In this study, seven initially considered variables were quickly screened with only 12 experimental runs and only three of them were detected to be actively contributing to modifying the responses under investigation. The optimisation of NLC was then obtained following a Box–Behnken design [116], as described later in Section 3.5.3.

### 3.5. Optimisation Designs

**Figure 5:**
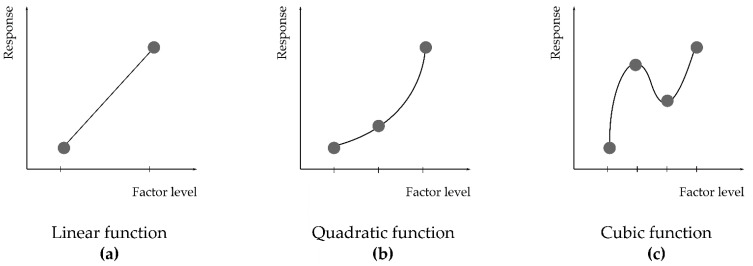
Graphical representation of the possible response function and the number of levels required for its estimation. Two-level factorial designs presuppose a linear response (**a**), while the inclusion of intermediate points permits one to calculate the possible curvature in the response (**b**,**c**).

Since screening designs only explore two levels of each factor, these designs allow one to model only linear functions (Figure 5a). Optimisation designs (also known as response surface methodologies), on the other hand, explore at least three levels of each factor and thus can more accurately address any lack of linearity by creating polynomials that contain quadratic (Figure 5b) and/or cubic terms (Figure 5c). The number of available experimental designs in this category is elevated, resulting in different possible valid approaches to the same optimisation quest [91].

Multiple outputs might be optimised simultaneously with a single experimental de-sign, obtaining for each response a different regression model. Simultaneous optimisation of various responses can be easily achieved through desirability functions, usually present in DoE software. The operator can select to maximise, minimise or set at a target value or range different responses of interest, and a possible solution that meets the criteria is calculated. As a result, new formulations with new factor combinations are calculated and proposed by the software, assigning each candidate a *desirability index* with a maximum value of 1 (perfect desirability) and 0 as an undesirable result. The higher this value is, the better the proposed factor combination results in a product that satisfies all the fixed criteria [117,118].

#### 3.5.1. Three-Level Factorial Designs

**Figure 6:**
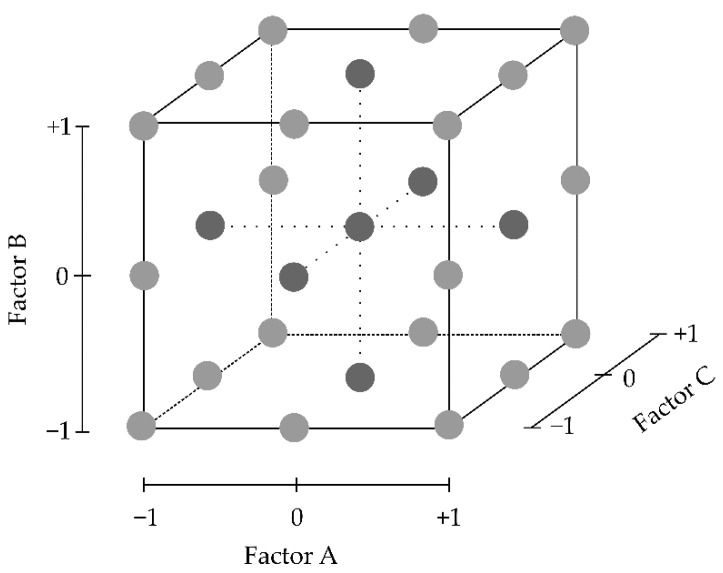
Geometric representation of a 3^3^ full factorial design with 27 experimental runs (grey dots). Note the existence of low (−1), intermediate (0) and high (+1) levels for each factor, leading to the existence of an overall centre-point run.

Three-level full factorial designs adequately estimate non-linear effects, but with in-creasing factors, the required experimental work increases exponentially. As an example, considering only four factors, a minimum of 81 runs (3^4^ = 81) is required if all the possible combinations are studied [108,119]. Two-level factorial designs explore combinations only at the vertices of the design space, while three-level factorial designs enable the exploration of factor combinations also at intermediate factor values (Figure 6).

Three-level full factorial approaches remain of interest only if two or three factors are under study, as demonstrated by the available literature in the field of interest in the present publication [120,121,122,123]. Youshia et al. [120] developed lipid-based nanocarriers loaded with the anti-glaucoma drug methazolamide containing a heterogeneous solid lipid matrix where the Compritol^®^ 888 ATO (glycerol behenate)-to-cetostearyl-alcohol ratio and also the surfactant concentration (Tween^®^ 80) were selected as factors in a 3^2^ full factorial design (Table 2).

Mixed-lipid matrices have been demonstrated to increase drug loading since the heterogeneous composition modifies crystallisation behaviour (See Section 2.2). Including the ratio between lipid components as a factor to be studied allows one to determine the optimum matrix composition to improve entrapment efficiency. Statistical analysis of the results revealed how increasing concentrations of the solid aliphatic alcohol allows for larger dissolution of slightly hydrophilic compounds such as methazolamide. No visual irritation and an increased pharmacological response were demonstrated after in vivo topical administration of the optimised formulation in albino rabbits when compared to a methazolamide solution. The presence of stearylamine, a surface-charge modifier, confers a positive charge, enhancing mucin interaction and ultimately increasing the retention time of the formulation. Similar results have been achieved with cetyltrimethylammonium bromide (CTAB), which Fangueiro and colleagues [121] used to confer a positive charge to a lipid nanoparticle with a water/oil/water structure containing Softisan^®^ 100 (lipid mixture), Lipoid S75 (soybean phosphatidylcholine) and Poloxamer 188. A three-factor three-level full factorial design (Table 2) demonstrated that the lecithin concentration in the formulation (Lipoid S75) was the key factor affecting nanoparticle size and the PDI (*p* < 0.05). Surface functionalisation with CTAB was studied after nanoparticle optimisation at varying concentrations of CTAB, analysing in vitro cytotoxicity due to CTAB addition. The optimised formulation containing CTAB (0.5% of the lipid phase) was stable over time and biocompatible, as demonstrated with Alamar blue assay in the human retinoblastoma cell line. Although blank formulations were tested, results appear promising for their use in ocular delivery. In fact, epigallocatechin gallate, a useful antioxidant agent in the treatment of several ocular diseases, was successfully loaded into the nanoparticles in a further publication of the same research group [124].

Other examples of the use of three-level factorial design are proposed for the development of brimonidine tartrate nanoemulsion [122] and bimatoprost-loaded SLN [123], as reported in Table 2.

It is important to underline that 3^k^ full or fractional factorials do not show rotatability. Rotatable designs provide the same precision in response estimations at all experimental points equidistant from the design origin. Rotatability is a desirable characteristic, especially if no previous information is available about the direction within the design space in which the optimal region will be found. Other response surface designs such as circumscribed central-composite or Box–Behnken designs can be rotatable, or *nearly* rotatable, and thus provide the same estimation quality on equidistant points from the centre of the design [91,93].

#### 3.5.2. Central Composite Designs (CCDs)

**Figure 7:**
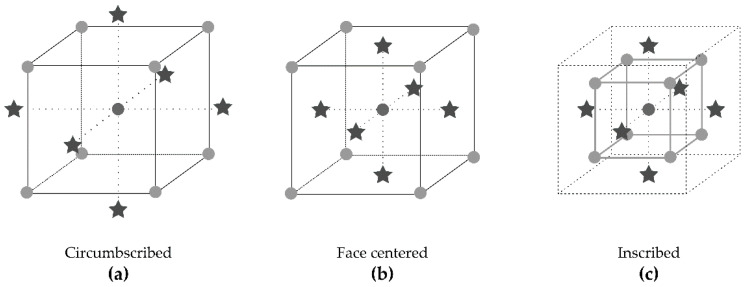
Graphical representation of the design space in three different typologies of the central composite designs s(CCDs). In circumscribed designs (**a**), the star points are set at an alpha distance from the centre point, which leads to new extreme high and low values for all studied factors. If alpha = ±1, star points fall on the centre of each face of the factorial space and a face-centred CCD is obtained (**b**). If the extremes values determined by a circumscribed design cannot be studied, an inscribed CCD (**c**) considers alpha points as limit values (alpha = ±1), and the initial factor settings are scaled down to fit within this new experimental domain.

Central composite designs are based on classical two-level full factorial designs to which new intermediate points have been added. These intermediate points, called *star points*, are located at an alpha distance from the design centre, which determines design rotatability (Figure 7) [91].

Three different typologies are possible considering alpha distance values: circum-scribed, face-centred and inscribed. Circumscribed and inscribed central composite designs (Figure 7a,c, respectively) require five levels of each factor and show rotatability, while face-centred CCDs (Figure 7b) require only three levels but are non-rotatable. It is also important to consider that circumscribed CCDs need to select factor settings at extreme values that might not be reachable for all factors. One advantage of these designs is their ability to augment pre-existing data from a two-level factorial design. By adding some star point runs, the new CCD allows for quadratic term estimation [91].

Several examples of CCDs applied to lipid nanoparticle optimisation found in the literature are reported in Table 3. Particularly, Yadav and colleagues [125] recently proposed a new approach for the treatment of age-related macular degeneration based on atorvastatin-loaded solid lipid nanoparticles intended to be topically administered. Preliminary studies allowed them to demonstrate that the concentration of all the four formulation components (lipid matrices and co-/surfactants) affected the responses under study. A central composite design (alpha = 1) concluded that the two responses of interest, particle size and entrapment efficiency, can be expressed as second-order functions of two independent variables: the Compritol^®^ 888 ATO/Phospholipon^®^ 90H and the Poloxamer 188/polyethylene glycol (PEG) 400 ratios (Table 3). The optimal formulation was obtained based on the criteria of minimising particle size while maximising entrapment efficiency. Further studies demonstrated improved atorvastatin stability, good ocular compatibility and enhanced transcorneal permeability in comparison to an atorvastatin suspension.

In a series of publications, González-Mira and colleagues explored the application of rotatable CCDs for anti-inflammatory flurbiprofen-loaded NLC optimisation. After a preliminary two-level full factorial design study [107] (Table 1), adequate lipid excipients were selected based on drug solubility and ocular compatibility criteria [126]. The three-factor, five-level rotatable CCD allowed, with only 16 experimental runs, them to create a regression model for each of the four responses under study (Table 3). The obtained optimised formulation showed promising physicochemical and colloidal characteristics for ocular administration, and ocular safety was assessed through the in vivo Draize test. Collected data allowed them to formulate new flurbiprofen-loaded NLC [127], where cetostearyl alcohol, previously used as a solid lipid matrix, was substituted with Compritol^®^ 888 ATO (Table 3). A further response was optimised, namely destabilisation time, that quantifies formulation sedimentation or creaming over the storage period (15 days, room temperature). Obtained NLC were included into a carbomer hydrogel in order to increase the formulation’s corneal retention time. The resulting semisolid formulation was rheologically characterised, and the ex vivo corneal permeation studies indicated that NLC inclusion into a hydrogel guarantees similar drug permeation than NLC alone. In any case, enhanced corneal permeation was observed when compared to a drug solution, and in vivo tests in New Zealand white rabbits further confirmed ocular tolerability.

Triamcinolone acetonide, a corticosteroid with both anti-inflammatory and antioedemous action, was included into NLC, as reported in a publication presented by Araújo and colleagues [128]. Nanoparticles, prepared for targeting the posterior segment of the eye by topical administration, were developed following an initial 2^4^ full factorial design, augmented with eight star points at alpha >1 distance (±1.68) and two centre points to ensure adequate quadratic term estimation. The resulting optimised formulation demonstrated nanoparticles with a spherical shape, with a particle size lower than 200 nm and a negative surface charge (see Table 3). Triamcinolone acetonide was entrapped into the amorphous matrix consisting of Precirol^®^ ATO 5 and squalene with Lutrol^®^ F68 as a surfactant. In a second publication [129], long-term stability was assessed, and in vivo studies on mice demonstrated that the same nanoparticles loaded with Nile Red (a fluorescent dye used instead of triamcinolone acetonide) can be detected at the posterior segment after topical administration.

Natural antioxidant agents, such as curcumin or quercetin, have also been selected to produce ophthalmic NLC [106,130] (Table 3). The use of lipid nanocarriers for antioxidant agent encapsulation appears suitable not only to increase solubility but especially to guarantee long-term stability. Particularly, optimisation of curcumin-loaded NLC formulations was achieved through a central composite design. Three material attributes were considered as numerical factors, and thus studied at three levels (face-centred CCD), while two processing parameters were studied as categorical factors at only two levels (Table 3). An optimised formulation with a desirability index of 0.977 (minimised particle size and PDI) was obtained with adequate stability for over three months when stored at 4 °C. Due to the low nanoparticle average size (approx. 70 nm) and the narrow PDI, a significant increase in curcumin permeation (∼2.5-fold) across the rabbit cornea in comparison to the control (curcumin propylene-glycol suspension) was demonstrated [106].

#### 3.5.3. Box–Behnken Designs (BBDs)

**Figure 8:**
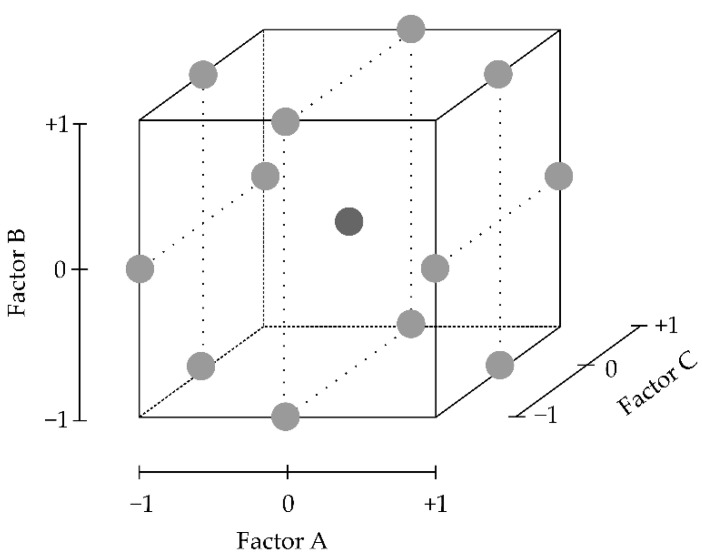
Graphical representation of the experimental runs for a three-factor Box–Behnken de-sign. Note that factor combinations happen at intermediate values (coded as “0”) for each factor, avoiding extreme value combinations.

Box–Behnken Designs (BBDs) were firstly introduced in the early 1960s [131]. As presented in Figure 8, the experimental runs do not contain an embedded full or fractional factorial, and all the experimental runs are at a middle-point value. In addition, only three levels for each factor are required. This design allows one to avoid combinations of factors at their extreme values, which might be useful, for example, when optimising processing parameters such as pressure and temperature that cannot reach extreme value combinations [132].

Another advantage is the reduced number of experimental runs required in comparison to other response surface methodologies. As an example, for three factors under study, CCDs would require a minimum of 20 runs and possibly new extreme factor settings (alpha points), while a BBD requires only 15 experimental runs and combinations of factor extremes are avoided. Nonetheless, this advantage disappears when the number of factors considered is higher than four [91,133].

A good number of examples are available in the literature for the optimisation of lipid-based nanocarriers, as depicted in Table 4 [116,134,135,136,137,138,139].

The first study here presented shows a sequential approach in which Rathod and colleagues [116] started with an initial Plackett–Burman experimental design (see Section 3.4) to develop ibuprofen-loaded NLC. Seven initial factors (amongst others, surfactant type and concentration, lipid concentration, homogenisation speed and time) were quickly screened in only 12 experimental runs by assessing their influence on three responses: particle average size, PDI and zeta potential. Only three factors demonstrated to influence the responses under study, which were further modelled via a Box–Behnken experimental design, including the drug entrapment efficiency as a fourth dependent variable. Quadratic effects were observed for three of the responses, while entrapment efficiency demonstrated to be not influenced by the variables under study. A robust optimised formulation, stable over 1 month if stored between 2 and 8 °C, was obtained with up to 3% of effectively loaded ibuprofen that showed no burst release and a sustained release over 12 h.

Following a three-factor Box–Behnken design, Baig and colleagues [134] prepared besifloxacin-loaded cationic nanostructured lipid carriers. Besifloxacin is a practically insoluble fourth-generation fluoroquinolone, approved as suspension by the FDA for the treatment of bacterial conjunctivitis [140]. Three independent variables regarding excipient concentrations were selected for the optimisation of nanoparticle size, PDI and zeta potential in only 17 runs. All three responses were fitted to quadratic models, which demonstrated adequate correlation values and a satisfactory lack-of-fit testing. To further increase drug bioavailability, cetyltrimethylammonium bromide (CTAB) was added for nanoparticle surface functionalisation. Cationic NLC (0.02% CTAB) demonstrated lack of toxicity when tested in vitro on porcine fibroblasts. In addition, when fluorescent rhodamine B was chosen as a loading agent, a significant fibroblast uptake was observed.

PEGylated NLC were also prepared for ophthalmic delivery of antimycotic agents (Table 4) [135,136]. Particularly, Patil and colleagues [136] selected the antifungal agent natamycin, available on the market as a suspension containing the drug in a micronised form (Natacyn^®^) [141] and approved for the treatment of fungal keratitis and endophthalmitis [142]. PEGylated natamycin-loaded NLC were obtained with the aim to improve the bioavailability of the antifungal drug. After an initial lipid-screening test, three critical material attributes and one process parameter were included in a Box–Behnken experimental design for optimisation of four responses (Table 4). Data analysis allowed authors to establish that the nanoparticle average size, drug loading and entrapment efficiency were dependent on the studied factors, while no statistical significance for the polydispersity index was found, indicating PDI robustness to factor settings. The optimal formulation was obtained with a desirability index of 0.9835, and in vitro studies demonstrated a transcorneal permeation improvement with respect to non-PEGylated NLC and natamycin suspension. The superiority of PEGylated nanoparticles was further demonstrated by in vivo studies on New Zealand white albino rabbits, since the natamycin concentration detected in the vitreous body appeared higher than the values observed for the drug suspension.

Transcorneal delivery of natamycin-loaded SLN was also studied by Khames and colleagues [137], who optimised the formulation by application of a Box–Behnken experimental design. A non-linear behaviour was demonstrated for each response under study (particle size, zeta potential and entrapment efficiency), and adequate quadratic models were proposed. Two formulations with a high desirability index of 0.953 and 0.949 were calculated by the software on the premises of minimising the particle size, maximising the drug entrapment efficiency and maintaining a zeta potential between 25 and 35 mV. The optimised formulation expressed an extended release over 10 h and increased ex vivo corneal penetration compared to natamycin saline suspension. Increased antifungal activity against *Aspergillus fumigatus* ATCC 1022 and *Candida albicans* clinical isolates compared to the plain drug was also demonstrated.

An optimised levofloxacin-based formulation (maximised entrapment efficiency and minimised particle size) showed sustained drug release and a comparable antibacterial (*S. aureus* and *E. coli*) in vitro activity compared to commercially available formulations [138].

Finally, Kalam and colleagues [139] proposed two different matrix compositions for the formulation of solid lipid nanoparticles (SLN) that contained gatifloxacin, a poorly soluble third-generation quinolone. Two simultaneous experimental designs allowed authors to optimise both formulations, which differed in the lipid matrix composition (Table 4). Five responses were fitted to quadratic models that quantified their influence on three variables under study. Optimised formulations (maximising entrapment efficiency and drug release while minimising particle size) showed good long-term stability, and the drug release pattern was found to follow a Korsmeyer–Peppas model. Ocular pharmacokinetic and in vivo safety were evaluated elsewhere [143].

### 3.6. Other Experimental Designs

The number of available experimental designs is extensive, but regarding research reported on lipid-based nanocarriers, two further peculiar typologies might be explained: mixture designs and Taguchi designs [93].

Mixture designs consider the studied factors to be the components of a blend [144]. The mathematical perspective of this approach is that the relative proportions between the components of a mixture must sum up to one. This fundamental constraint leads to one limitation: increments in one component lead to diminution in the proportion of at least one of the other components. The measured response, therefore, is not supposed to depend on the quantity of each component (the quantity of mixture) but on the relative proportions between each of them [145]. A natural approach is considering that all factor blends are possible and thus the relative proportions of each component can freely range from zero to unity. In this case, boundary designs such as simplex-lattice or simplex-centroid mixture designs propose experimental runs based on single-component mixtures, located on the vertexes, and binary mixtures, located on the edges of the factor space. The overall centroid point contains all mixture components in equal proportions. Axial designs, on the other hand, place the experimental runs inside the simplex region, and only *complete* mixtures are studied. In pharmaceutical formulations, in most cases, only complete mixtures will be of interest, so research time and model efficiency might be improved by individualising a constrained subregion of interest within the whole simplex. Setting upper and/or lower boundaries to at least one of the components leads to the so-called extreme vertices designs, allowing for a more detailed investigation of the region of interest. It is important to note that in most cases, even though a mixture design *should* be considered, the same problem can be proposed as a factorial design, as explained by Cornell in [146].

Interpretation of the effects might become difficult if more than three mixture components are present, so it can be helpful to virtually consider similar excipients as a single mixture component. In this sense, lipid-based nanocarrier excipients can be formulated as the components of a pseudo-ternary mixture of water, lipids and surfactants-cosurfactants [147,148]. If some upper and/or lower boundaries are known for one or more of the components, constrained mixture designs allow to explore only a subset of the design space [149]. In case the number of factors is elevated, screening mixture designs allow to select the vital few contributing to the responses under study [150]. Similarly, process variables can also be included in the so-called mixture-process variable designs. Moreover, it is possible to create mixture-amount designs, where the amount of mixture is also considered a factor under study [146,151].

Shah and colleagues [152] developed a nanoemulsion-based vehicle for moxifloxacin ocular delivery following a mixture design. The pseudo-ternary mixture consisted of water, oil (ethyl oleate) and a mixture of surfactants (Tween^®^ 80 and Soluphor^®^ P). The only response to be optimised in the mixture design was the nanoemulsion particle size. The second-order model demonstrated that the relationship between the proportion of water and surfactants did not affect the particle size, and drug loading reached 0.5% *w/w*. The resulting optimised formulation, stable upon dilution, guaranteed antibiotic therapeutic concentrations in the aqueous humour when administered in vivo to albino rabbits.

Genichi Taguchi proposed a new conception of experimental designs, resulting in the creation of the so-called *Taguchi designs* or *orthogonal arrays*. Though based on classical factorial designs, Taguchi designs are of help in factor-screening stages and for process robustness analysis [91,153,154]. As an example, Wang and colleagues [155] reported novel chitosan-coated SLN for topical administration containing methazolamide, a potent antiglaucoma drug, that had been optimised through two consecutive experimental designs. A first factor screening was performed using a five-factor, four-level orthogonal design where the five factors evaluated were methazolamide, Lipoid S100 and glyceryl monostearate content and also the percentage of co-emulsifiers and chitosan concentration. Three of those factors (namely, Lipoid S100, glyceryl monostearate and chitosan) demonstrated to actively modify the responses of interest (nanoparticle average size, zeta potential, drug loading and entrapment efficiency), being further optimised in a three-factor Box–Behnken experimental design. The obtained chitosan SLN (247.7 ± 17.3 nm; 33.5 ± 3.9 mV) showed improved intraocular pressure reduction in in vivo studies on albino rabbits in comparison to uncoated SLN.

### 3.7. Data Analysis and Model Application

The obtained response values for each experimental run must undergo the critical step of data analysis, which has been mainly simplified with current available DoE software [156]. The overall aim is to obtain a regression model that accurately predicts the response by conferring an adequate regression coefficient to each main effect and interaction included in the polynomial. Equally important is to develop the lowest-order model with few terms that still accurately predicts the response. This is achieved by multiple linear regression, which at the same time requires least-squares analysis.

Initial inspection of raw experimental data can serve as a starting point for the next stage of regression analysis. Multiple statistical tools are available: analysis of variance (ANOVA), R-squared (R^2^) values, normal probability plots and residual plots, amongst others. The R^2^ value is an important indicator of the level at which the obtained model fits the raw data (*goodness of fit*), and the more its value approaches 1, the better. However, if interpreted alone, it can lead to false conclusions, and a further indicator is required. Predicted-R^2^ is an indicator of the model *goodness of prediction* and accounts for its ability to predict responses for new, previously unexplored factor settings. A model can show high R^2^ values while showing negative values for predicted-R^2^, indicating that the model has no predictive power, though fitting the initial data (it is predicting experimental noise) [92].

The analysis of variance (ANOVA), in combination with Fisher tests, helps to evaluate model significance and lack of fit, both indicators serving as a statistical validation of the polynomials generated by DoE software. The proposed model is statistically significant if *p* < 0.05 (at a 95% level of significance), and if enough replicates have been included, the lack-of-fit test should be *p* > 0.05, otherwise the model is explaining random noise. Further graphical tools also allow to detect whether any response transformation is required and to check for outliers [90,110]. The obtained polynomial can be plotted, as shown in Figure 3, for a better understanding of factor contribution to the response.

Once the analysis step is concluded with a satisfactory model equation, its validity can be further assessed via confirmatory runs. New trials with unexplored factors settings are performed, and the response experimental data are compared to the predicted response of the model.

Finally, current DoE software packages include optimisation tools that use the obtained regression models to find the best factor combination that leads to desired product specifications. The working principle is based on desirability functions that allow one to simultaneously optimise multiple responses, as explained in Section 3.5 [117,118]. The researcher can select whether each response is desired to be minimised, maximised or set in a range, and the software proposes multiple solutions (expressed as factor combinations) with predicted responses that meet to in a greater or lesser extent the pre-established criteria. Each solution is assigned a desirability index, which is highly dependent on the severity of the optimisation criteria established by the operator. As an example, the besifloxacin optimised nanoformulation in Table 4 was calculated with a desirability index of 0.278, indicating poor similarity to the pre-established criteria. Better values were obtained for natamycin-loaded optimised NLC with a desirability index of 0.953 with the premises of setting the zeta potential in a range between 25 and 35 mV, minimising the nanoparticle average size and maximising the entrapment efficiency. The desirability functions can also be plotted in order to identify their variation across the design space.

## 4. Conclusions and Perspectives

The purpose of this review was to highlight the latest trends in optimisation of lipid-based nanocarriers intended for ocular administration by the statistical design of experiments. It is clear from the reported research that development of mathematical regression models that describe the lipid nanoparticulate production process helps in identifying critical factors controlling crucial characteristics of the colloid, such as the nanoparticle average size, zeta potential and entrapment efficiency. Implementation of quality-by-design (QbD) production approaches could especially benefit market access of nanosystems, and the design of experiments is a key tool in this process, which can already be implemented in early research stages.

The presented research on lipid-based nanocarriers has also shown that one optimisation problem can be approached following different experimental designs, still arriving, in any case, at valid polynomials [97]. For instance, the same formulation problem regarding solid lipid nanoparticles has been optimised by a three-level full factorial, central composite, Box–Behnken or Taguchi designs [120,125,134,155]. It is important to underline that one single experimental design will in most cases not offer all the responses and that meticulous planification and statistical analysis are more important than experimental work. Sequential approaches, with adequate pre-experimental work, have an increased chance of successful DoE. This is evident from the reviewed publications where early lipid-solubility-screening tests allowed researchers to select the best nanoparticle excipients, which ultimately resulted in improved entrapment efficiency values. Since response values are the main tool for model regression establishment, it is important to underline the necessity to employ low-variable and highly reliable measuring techniques as well as devices that are in a good calibration state.

The current available literature lacks full and complete quality-by-design approaches to general pharmaceutical production. Poor familiarity with the statistical tools and the required theoretical background and work planification might be the main reasons for this issue [13,157]. Nonetheless, some approved and marketed products, not yet related to ophthalmic diseases or nanotechnology, have included QbD approaches during their application process, such as Kalydeco^®^, Gazyvaro^®^, Januvia^®^ or Gazyva^®^ [158,159,160,161,162]. However, full quality-by-design approaches might be cumbersome for an initial research stage, but familiarisation with DoE can be a good starting point towards more effective research.

Some decades ago, effective DoE implementation would have required highly specialised mathematical skills for data analysis, but nowadays, several user-friendly tools are easily available to researchers [156]. However, since experimental choices are driven by the researcher and not by the software, a good theoretical basis in statistics is required to avoid inadequate data interpretation. In fact, it is important to keep in mind the possible bias in some of the results reported above. Some strategies such as experimental run randomisation or trial replicates for increasing model degrees of freedom might have not been adequately applied in some cases. Appropriate knowledge of the prediction capability of each experimental design, its resolution or level of confounding is also important. Insufficient planning or avoiding factor-screening stages might lead to neglecting crucial factors with a consequent useless regression model, losing time and money.

Considering the application to the ocular route, the authors acknowledge the lack of in vitro, ex vivo or in vivo tests in some publications, which could have further reinforced the adequacy of DoE inclusion in the process of developing suitable ophthalmic products. An interesting discussion on the barriers hindering widespread application of DoE, offered by A. Jiju, can be found in [90].

Regarding the focus of the present review, other lipid-containing nanoparticulate systems, such as cubosomes [163] or niosomes [164], could have been considered but would fall out of the scope of this review. Mathematical and statistical terms might have been oversimplified in some cases for better concept comprehension. As stated before, we have hereby presented a non-exhaustive compilation of available experimental designs, focusing on the available examples of application in the ophthalmic lipid-based nanocarriers field.

Further research in this field might lead to inclusion of different experimental designs and an extensive diffusion of DoE in the research community. Moreover, DoE might just be the inception towards the application of more complex computational methods such as artificial intelligence for drug delivery improvement [165,166,167,168]. In fact, promising results have already been reached by applying artificial intelligence in the optimisation of single CQAs, such as the drug entrapment efficiency [169], or whole NLC-based end products [170].

## Figures and Tables

**Table 1:** Examples of the application of two-level (2k) full factorial designs in lipid-based nanocarrier production.

Loaded Agent	Pre-Experimental Studies	Independent Variables	Dependent Variables	Improved Formulation (±SD)	Ref.
Dexamethasone	Drug-excipient solubility screening Matrix crystallinity studies (DSC, XRD)	Dexamethasone (% *w/w*) Surfactant (% *w/w*) Lipid (% *w/w*)	Size PDI ZP %EE	Size: 92.18 ± 0.49 nm PDI: 0.12 ± 0.02 ZP: −7.62 ± 0.26 mV %EE: 88.31%	[113]
Dexamethasone	Data obtained in [113] employed	Dexamethasone (% *w/w*) Surfactant (% *w/w*) Mucoadhesive polymer (% *w/w*)	Size PDI ZP %EE d(0.5) Span value	Size: 200.70 ± 7.63 nm PDI: 0.34 ± 0.039 ZP: −7.4 ± 0.1 mV %EE: 86.94% d(0.5): 0.122 Span value: 1.416	[114]
Flurbiprofen	Lipid solubility screening Matrix crystallinity studies (DSC)	Flurbiprofen (% *w/w*) Tween^®^ 80 (% *w/w*) Stearic acid (% *w/w*) regarding total lipid Storage temperature (°C)	Size PDI ZP	Size: 288 ± 10.6 nm PDI: 0.245 ± 0.013 ZP: −29.0 ± 0.557 mV Storage temp.: 25 °C %EE: 92.76 ± 2.54%	[107]

Abbreviations: d(0.5), volume median diameter; DSC, differential scanning calorimetry; PDI, polydispersity index; span value, width of nanoparticle volume distribution; XRD, X-ray diffractometry; ZP, zeta potential; % *w/w*, weight concentration; %EE, drug entrapment efficiency as a percentage.

**Table 2:** Examples of the application of 3-level full factorial designs in the production and optimisation of lipid-based nanocarriers.

Particle Type	Loaded Agent	Pre-Formulation Studies	Independent Variables	Dependent Variables	Optimal Formulation (±SD)	Ref.
Cationic SLN	Methazolamide	Drug solubility assay in heterolipid mixtures (data not shown)	Cetostearyl-alcohol-to-glycerol-behenate ratio (% *w/w*) Tween® 80 (% *w/w*)	Size PDI ZP %EE	Size: 207.1 ± 6.45 nm PDI: 0.243 ZP: 41.50 ± 0.33 mV EE%: 25.62 ± 0.89%	[120]
Lipid nano-Particle (*w/o/w*)	N/a	N/a	Softisan® 100 (% *w/w*) Lipoid S75 (% *w/w*) Poloxamer 188 (% *w/w*)	Size PDI ZP	Size (0.5% CTAB): 194.4 ± 0.43 nm PDI: 0.185 ± 0.02 ZP: +37.20 ± 1.27 mV	[121]
Nano-emulsion	Brimonidine tartrate	Excipient FT-IR, DSC and XRD studies	Castor oil (% *w/w*) Lipoid-S75-to-Lipoid-E80 ratio (% *w/w*) Pluronic® F68 ^a^ (% *w/w*)	Size PDI %EE	Size: 272.7 nm PDI: 0.270 %EE: 79.25 ± 2.85%	[122]
SLN (in situ gel)	Bimatoprost	Lipid with a melting point about 50–55 °C selection	Glyceryl monostearate (mg) Tween® 80 (% *w/w*)	Size PDI ZP %EE	Size: 148.4 ± 1.25 nm PDI: 0.156 ± 0.04 ZP: −19.3 ± 1.40 mV EE%: 83.5 ± 0.27%	[123]

^a^ Pluronic^®^ F68 = Poloxamer 188. Abbreviations: CTAB, cetyltrimethylammonium bromide; DSC, differential scanning calorimetry; FT-IR, Fourier-transform infrared spectroscopy; N/a, data not available/shown; PDI, polydispersity index; XRD, X-ray diffractometry; ZP, zeta potential; % *w/w*, weight concentration; %EE, drug entrapment efficiency expressed as a percentage.

**Table 3:** Examples of the application of central composite designs in the production and optimisation of lipid-based nanocarriers intended for ocular delivery.

Particle Type	Loaded Agent	Pre-Formulation Studies	Independent Variables	Dependent Variables	Optimal Formulation (±SD)	Remarks	Ref.
SLN	Atorvastatin	·Surfactant- and solvent-pre-screening studies ·Initial formulation trials for factor level and process parameters selection	**Set at 3 levels**: Poloxamer-188-to-PEG-400 ratio ·Compritol^®^-888-ATO-to-Phospholipon^®^-90H ratio	Size %EE	Size: 256.3 ± 10.5 nm PDI: 0.26 ± 0.02 %EE: 73.1 ± 1.52%	·Improved drug stability Ocular safety Increased ex vivo corneal permeability vs. drug suspension	[125]
NLC	Flurbiprofen	Lipid-screening tests Selection of critical variables affecting the response	**Set at 5 levels:** Oil concentration in the total lipid phase (%) Tween^®^ 80 (%) Flurbiprofen (%)	Size PDI %EE 90%LD	Size: 228.3 ± 4.2 nm PDI: 0.156 ± 0.017 %EE: 89.4 ± 0.7% 90%LD: 0.303 ± 0.098 μm	·Sustained release (Korsmeyer–Peppas model) In vivo ocular safety	[126]
NLC-based hydrogel	Flurbiprofen	Already performed in [126]	**Set at 5 levels:** ·Oil concentration in the lipid phase (% *w/w*) ·Tween^®^ 80 (% *w/w*) ·Flurbiprofen (% *w/w*)	Size PDI ZP %EE Destabilisation time	·Size: ≤199 ± 0.003 nm ·PDI: 0.152 ± 0.017 ·ZP: −23.0± 0.569 mV ·%EE: 88.7± 0.6%	In vivo ocular safety Increased ex vivo corneal permeability vs. flurbiprofen solution	[127]
NLC	Triamcinolone acetonide	·Lipid-screening tests ·Process parameter evaluation through preliminary experiments	**Set at 5 levels:** ·Lipid phase (%) ·Precirol^®^ ATO 5 in the lipid phase (%) ·Lutrol^®^ F68 ^a^ (%) ·Triamcinolone acetonide (%)	Size PDI ZP %EE	·Size < 200 nm ·PDI: ∼0.1 ·ZP: ∼ −45 mV ·%EE: ∼95%	·Spherical nanoparticle shape ·In vivo ocular safety	[128]
NLC	Curcumin	·Lipid screening for drug solubility and stability	**Set at 3 levels:** ·Poloxamer 188 (%) ·Vitamin E TPGS ^b^ (%) ·Olive oil (%) Set at 2 levels (categorical): ·Homogenisation (rpm) ·Sonication time (min)	Size PDI	·Size: 66.8 ± 2 nm ·PDI: 0.17 ± 0.05 ·%EE: 96 ± 1.6% ·Drug loading: 3.1 ± 0.05%	Improved curcumin stability ·Increased ex vivo corneal permeability vs. curcumin suspension	[106]
NLC-based hydrogel	Quercetin	·Preliminary tests for critical factor detection ·Excipient DSC studies	**Set at 5 levels:** ·Quercetin (% *w/v*) ·Oil (% *w/v*) ·Cremophor EL (% *w/v*)	Size PDI %EE	·Size: ∼75.54 nm ·PDI: ∼0.180 ·%EE: ∼97.14%	Adequate sol-gel transition (<35 °C; pH 7.4) ·Sustained release of ∼80% quercetin over three days of the NLC-based hydrogel (carboxymethyl chitosan/Poloxamer 407)	[130]

^a^ Lutrol^®^ F68, Poloxamer 188. ^b^ Vitamin E TPGS, tocopherol polyethylene glycol succinate. Abbreviations: 90%LD, average diameter (volume distribution) that 90% of the particles express; DSC, differential scanning calorimetry; PDI, polydispersity index; ZP, zeta potential; % *w/w*, weight concentration; % *w/v*, mass concentration per volume; %EE, drug entrapment efficiency expressed as a percentage.

**Table 4:** Examples of the application of Box–Behnken designs in the production and optimisation of lipid-based nanocarriers intended for ocular delivery.

Particle Type	Loaded Agent	Pre-Formulation Studies	Independent Variables	Dependent Variables	Optimal Formulation (±SD)	Desirability Index	Ref.
NLC	Ibuprofen	·Lipid- and surfactant-screening studies Plackett–Burman design	·Surfactant (% *w/w*) ·Lipid (% *w/w*) ·Ratio of surfactants	Size PDI ZP EE%	Size: ∼147 nm PDI: ∼0.159 ZP: ∼−25.7 mV EE%: ∼97.89%	Not reported	[116]
Cationic NLC	Besifloxacin and rhodamine B	·Initial trials for excipient nature and concentration selection	·Gelucire^®^ 50/13 (mg/mL) ·Compritol^®^ 888 ATO (mg/mL) ·Labrafac^®^ PG (µL/mL)	Size PDI ZP	Size: ∼173.6 nm PDI: ∼0.188 ZP: ∼16.6 mV EE%: ~80%	0.278 Maximise ZP Minimise PS Minimise PDI	[134]
PEGylated NLC	Amphotericin B	·Lipid- and PEG-screening tests	·DSPE-PEG−2000 ^a^ (% *w/v*) ·Amphotericin B (% *w/v*) ·Castor oil (% *w/v*) ·Cycles of HPH	Size PDI ZP EE% Drug loading	Size: 218 ± 5 nm PDI: 0.3 ± 0.02 EE%: 92.7 ± 2.5% Drug loading: 4.6 ± 0.1	0.9 Maximise EE% Maximise drug loading	[135]
Pegylated NLC	Natamycin	Lipid = screening study	·Castor oil (% *w/v*) ·Precirol^®^ ATO 5 (% *w/v*) ·Span 80 (% *w/v*) HPH time (min)	Size ZP EE% Drug loading %	Size: ∼241 nm PDI: ∼0.406 %EE: ∼95.35 Drug loading: ∼6.5%	0.9835 PS < 300 nm Minimise PDI · Maximise %EE · Maximise drug loading	[136]
SLN	Natamycin	·Lipid and surfactant solubility screening · Initial trials for factor-level selection	·Precirol^®^ ATO 5 (% *w/w*) ·Pluronic^®^ F68 (% *w/w*) ·Sonication frequency (kHz)	Size ZP %EE	Size: ∼42 nm ZP: ∼26 mV EE%: ~85%	0.953 ZP = 25–35 mV Minimise PS Maximise %EE	[137]
SLN	Levofloxacin	·Solubility screening in solvents and lipids ·Initial trials for factor-level selection	·Stearic acid (% *w/w*) ·Tween^®^ 80 (% *w/w*) ·Sodium deoxycholate (% *w/w*)	Size %EE	Size: ∼237.82 nm PDI: ∼0.251 EE%: ∼78.71%	Not reported	[138]
SLN	Gatifloxacin	Not reported	·Stearic acid and Compritol^®^ 888 ATO mixture (% *w/w*) ·Poloxamer 188 (% *w/w*) ·Sodium taurocholate and Transcutol^®^ P mixture (% *w/w*)	Size EE% % Drug released	Size: 251.4 ± 7.4 nm PDI: 0.338 ± 0.11 ZP: +29.5 ± 2.8 mV EE%: 78.55 ± 3.41 % Drug released: 84.24 ± 2.9	Not reported	[139]
			·Stearic acid and Gelucire^®^ 50/13 mixture (% *w/w*) ·Poloxamer 188 (% *w/w*) ·Sodium taurocholate and ethanol mixture (% *w/w*)	Size EE% % Drug released	Size: 297.2 ± 8.5 nm PDI: 0.268 ± 0.09 ZP: +30 ± 3.2 mV EE%: 46.58 ± 2.25 % Drug released: 79.23 ± 2.5		

^a^ DSPE-PEG-2000, 1,2-distearoyl-sn-glycero-3-phosphoethanolamine-N-(methoxy(polyethylene glycol)). Abbreviations: PEG, polyethylene glycol; DSC, differential scanning calorimetry; HPH, high-pressure homogenisation; PDI, polydispersity index; PS, nanoparticle average size; ZP, zeta potential; % *w/w*, weight concentration; % *w/v*, mass concentration per volume; %EE, drug entrapment efficiency expressed as a percentage.

## Data Availability

Data sharing not applicable.
